# Unveiling the bioactive potential of *Actinomycetota* from the Tagus River estuary

**DOI:** 10.1007/s10123-024-00483-0

**Published:** 2024-01-18

**Authors:** José Diogo Neves dos Santos, Eugénia Pinto, Jesús Martín, Francisca Vicente, Fernando Reyes, Olga Maria Lage

**Affiliations:** 1 Department of Biology, Faculty of Sciences, University of Porto, Rua Do Campo Alegre, S/N, 4169-007 Porto, Portugal; 2 Interdisciplinary Centre of Marine and Environmental Research, Terminal de Cruzeiros Do Porto de Leixões, University of Porto, Avenida General Norton de Matos, S/N, 4450-208 Matosinhos, Portugal; 3 Laboratory of Microbiology, Department of Biological Sciences, Faculty of Pharmacy, University of Porto, Rua Jorge de Viterbo Ferreira 228, 4050-313 Porto, Portugal; 4 Centro de Excelencia en Investigación de Medicamentos Innovadores en Andalucía, Fundación MEDINA, Avenida del Conocimiento, 34 Parque Tecnológico de Ciencias de La Salud, 18016 Granada, Spain

**Keywords:** iChip, Novel *Actinomycetota*, Antimicrobial activities, HPLC/HRMS, Marine *Actinomycetota*

## Abstract

The increase in global travel and the incorrect and excessive use of antibiotics has led to an unprecedented rise in antibiotic resistance in bacterial and fungal populations. To overcome these problems, novel bioactive natural products must be discovered, which may be found in underexplored environments, such as estuarine habitats. In the present work, estuarine actinomycetotal strains were isolated with conventional and iChip techniques from the Tagus estuary in Alcochete, Portugal, and analysed for different antimicrobial bioactivities. Extracts were produced from the isolated cultures and tested for bioactivity against *Staphylococcus aureus* ATCC 29213, *Escherichia coli* ATCC 25922, *Aspergillus fumigatus* ATCC 240305, *Candida albicans* ATCC 10231 and *Trichophyton rubrum* FF5. Furthermore, bioactive extracts were subjected to dereplication by high-performance liquid chromatography (HPLC) and high-resolution mass spectrometry (HRMS) to putatively identify their chemical components. In total, 105 isolates belonging to 3 genera were obtained. One which was isolated, MTZ3.1^ T^, represents a described novel taxon for which the name *Streptomyces meridianus* was proposed. Regarding the bioactivity testing, extracts from 12 strains proved to be active against *S. aureus*, 2 against *E. coli*, 4 against *A. fumigatus*, 3 against *C. albicans* and 10 against *T. rubrum*. Dereplication of bioactive extracts showed the presence of 28 known bioactive molecules, 35 hits have one or more possible matches in the DNP and 18 undescribed ones. These results showed that the isolated bacteria might be the source of new bioactive natural products.

**Supplementary Information:**

The online version contains supplementary material available at 10.1007/s10123-024-00483-0.

## Introduction

Humanity faces an increased risk from transmittable diseases, predominantly by the unprecedented increase in antibiotic resistance in bacterial and fungal populations, which is due to the abusive and inappropriate use of antibiotics and by a great expansion of global travel (Van Boeckel et al. 2019). These infections by resistant microorganisms have high morbidity and mortality, are more expensive to treat, and result in longer hospitalizations, placing undue burdens on healthcare systems (Frost et al. 2019). The World Health Organization (WHO) in the Global Antimicrobial Resistance Surveillance System (GLASS) report (WHO 2014, 2019) specifies that carbapenem-resistant *Enterobacteriaceae* and methicillin-resistant and/or vancomycin-resistant *Staphylococcus aureus* are top priorities for research and development of novel antibiotics. Regarding fungi, reports on *Candida* spp. and *Aspergillus* spp. are increasingly worrisome, with 90% of *Candida auris* isolates being resistant to fluconazole and 33% to amphotericin B (Lockhart et al. 2017). Azole resistance in *Aspergillus* is also being widely observed (Howard et al. 2009; Bueid et al. 2010; Pfaller 2012). In fact, the World Health Organization recently classified these microorganisms, alongside *Cryptococcus neoformans*, as the critical priority group for the development of novel treatments (WHO 2022).

To cope with these problems, novel bioactive natural products are needed, which may be found in underexplored environments, such as estuarine habitats. Estuaries are partially enclosed coastal water bodies located at the mouth of rivers where freshwaters from their stream mix with salt water from the ocean (McLusky 1989), forming a brackish environment with very high biomass productivity (Correll 1978). Besides being important in human societal development, these environments are important natural habitats for wildlife where organisms live, feed and reproduce (Gogina et al. 2018). Geographically, the Tagus estuary, formed at the junction of the biggest river in the Iberian peninsula and the western Atlantic coast of the same peninsula, is the largest estuary in Portugal with a wet area of 320 km^2^ at high tide and 130 km^2^ at low tide (Dias and Marques 1999). Three important regions in this estuary are as follows: the Tagus Estuary Natural Reserve, the largest natural reserve in Western Europe and an important life sanctuary (Cabral et al. 2001; Catry et al. 2011), a large water basin named Sea of Straw and the Rosário salt march. The total bacterial activity of these locations is due to bacterial concentrations of 2.0–53.5 × 10^9^ cells.L^−1^ in the salt marsh and of 1.8–13.1 × 10^9^ cells.L^−1^ in the channel water (Santos et al. 2007).

This immense microbial diversity may be a great reservoir of novel natural products (NPs). NPs of marine bacterial origin are now seen as particularly important in the drug discovery process, such as antimicrobials in particular (Santos et al. 2020). An upward trend is being seen in the number of discovered molecules and the remarkable chemical diversity displayed (Blunt et al. 2018; Carroll et al. 2020, 2023; Petersen et al. 2020). Marine bacterial NPs range from peptides, siderophores and polyketides to esters, macrolactones, quinones and terpenes, highlighting the bacterial potential for the discovery of novel active principles (Carroll et al. 2020). For example, novel carbon skeletons like Taromycin B (Reynolds et al. 2018) and janthinopolyenemycins (Anjum et al. 2018) show potent activity against methicillin-resistant *S*. *aureus* and vancomycin-resistant *Enterococcus faecium* and against *Candida albicans*, respectively.

*Actinomycetota* constitutes one of the most valuable bacterial phyla regarding the discovery of NPs (Blunt et al. 2018; Carroll et al. 2019, 2020; Santos et al. 2020). These bacteria are Gram-positive, aerobic and nonmotile and usually have genomes with a high G + C content. Morphologically, *Actinomycetota* can range from spore-forming aerial mycelium, such as *Streptomyces* spp. and *Micromonospora* spp., to asporogenous rods, for example *Gordonia* spp., and cocci, for instance *Micrococcus* spp. (Parte 2013, 2018; Parte et al. 2020). Ecologically, *Actinomycetota* are ubiquitous in most natural habitats, often having a vital role in the health of the environment. This is in part due to their ability to act as degraders of organic material (McCarthy and Williams 1992), to their participation in biogeochemical cycles, namely nitrogen and metal cycles (Bhatti et al. 2017), but also to their capacity to produce antimicrobials, modulating, thus, the microbial communities in the ecosystems. *Actinomycetota* and, in particular, the *Actinomycetales* are responsible for the production of over two-thirds of naturally derived antibiotics (Bentley et al. 2002) and novel bioactive molecules isolated over the past 20 years (Carroll et al. 2020; Santos et al. 2020). In fact, Taromycin B was isolated from the marine actinomycete *Saccharomonospora* sp. CNQ-490 (Reynolds et al. 2018).

The isolation of *Actinomycetota* can be achieved through various methods. These may include physical and chemical treatments and specific nutrient media. Physical methods include high temperatures or dry conditions to kill vegetative cells while leaving spores intact. Chemical methods use molecules, such as antibiotics, to control the growth of fast-growing Gram-negative bacteria and fungi (Jensen et al. 2005; Maldonado et al. 2005; Mincer et al. 2005). Nutritional media can be adjusted to include complex sugars and polymers which promote growth of *Actinomycetota* (Manivasagan et al. 2013; Bhatti et al. 2017). However, isolating new marine *Actinomycetota* can be difficult as they may be adapted to unique conditions in the ocean, implying different isolation approaches. One of these approaches is the isolation chip (iChip), which allows for an in situ enrichment of cells, thus facilitating the “domestication” and isolation of new strains (Nichols et al. 2010; Berdy et al. 2017). The iChip is a method that uses a plate with multiple wells, each containing a gelled suspension of environmental bacteria and covered by porous membranes, creating miniature diffusion chambers for the cells to grow under their natural conditions, particularly regarding their nutritional requirements.

As the prevalence of *Actinomycetota* in the drug discovery process is relevant, in this study, sporogenous actinomycetotal strains were isolated by both conventional isolation techniques and by simulating in situ conditions using an iChip-like approach (Santos et al. 2022; Vitorino et al. 2022). The sampling location chosen was in Alcochete, which is on the south side of the estuary near the Tagus Estuary Natural Reserve, an important biodiverse reserve and the largest wetland in Portugal. Samples of the water column, sediments and the alga *Ulva* sp. were collected. Isolates were identified based on 16S rRNA gene analysis and their extracts were analysed for antimicrobial activity against *Escherichia coli* ATCC 25922, *Staphylococcus aureus* ATCC 29213, *Aspergillus fumigatus* ATCC 240305, *Candida albicans* ATCC 10231 and *Trichophyton rubrum* FF5. Bioactive extracts were dereplicated by high-performance liquid chromatography (HPLC) and high-resolution mass spectrometry (HRMS) to putatively identify their chemical components.

## Methodology

### Sampling and isolation

During May 2021, at low tide, algal, sediment and water samples were collected at the pontoon of Alcochete, in the district of Setubal (38° 45′ 24.9″ N 8° 57′ 58.9″ W), part of the Sea of Straw. The water temperature, 20 °C, was measured using the temperature probe from an HI 8424 microcomputer pH meter (HANNA instruments) and salinity, 1.54%, using the salinity probe of a Multi 350i/SET (WTW, Germany). The samples were transported to the laboratory in a cold chamber. The processing of the samples was performed in several different ways. For the conventional isolation, all samples were heated at 55 °C for 30 min to kill vegetative cells, leaving behind only spores. Additionally, prior to this treatment, algal fragments were washed with sterile, filtered water from the sampling site. Afterwards, 200 µL from the heat-treated samples (water, sediment or alga) were inoculated on plates with 20 mL of modified M13 medium (0.25% w/v peptone, 0. 25% w/v yeast extract, 5 mM Tris–HCl pH 7.5, 0. 25% w/v glucose, 0.1% v/v of vitamin solution (0.1 µg.mL^−1^ cyanocobalamin, 2.0 µg.mL^−1^ biotin, 5.0 µg.mL^−1^ thiamine-HCl, 5.0 µg.mL^−1^ Ca-pantothenate, 2.0 µg.mL^−1^ folic acid, 5.0 µg.mL^−1^ riboflavin and 5.0 µg.mL^−1^ nicotinamide) and 0.2% v/v of Hutner’s solution (99 mg.L^−1^ FeSO_4_.7H_2_O, 12.67 mg.L^−1^ NaMoO_4_.2H_2_O, 3.34 g.L^−1^ CaCl_2_.2H_2_O, 29.70 g.L^−1^ MgSO_4_.7H_2_O, 50 mL.L^−1^ “44” metals solution and 10.0 g.L^−1^ nitrilotriacetic acid; for 100 mL of “44” metals: 250 mg ethylenediaminetetraacetic acid, 1095 mg ZnSO_4_.7H_2_O, 500 mg FeSO_4_.7H_2_O, 154 mg MnSO_4_.H_2_O, 39.2 mg CuSO_4_.5H_2_O, 24.8 mg Co(NO_3_)_2_.6H_2_O and 17.7 mg Na_2_B_4_O_7_.10H_2_O)) (Lage and Bondoso 2011) supplemented with nalidixic acid (300 mg/L) and cycloheximide (20 mg/L) and incubated at 25 °C. Colonies were allowed to grow for a period of 1 month, and the grown isolated colonies were then re-streaked onto fresh modified M13 medium agar plates and designated MTZ# (# = origin of the sample; 1, from sediment, 2 from *Ulva* sp., 3 from water column). For the iChip approach, a modified methodology from Santos et al. (2022) was used. As done previously, to replicate the miniature diffusion chambers present in the iChip culturing system (Nichols et al. 2010), a MultiScreen® 96-Well Filtration Plate was used which had a 0.22 µm filter on the underside. Around 10 mL of sediments was added to a falcon tube and 10 mL of sterile, filtered water from the sampling site was added and heated for 30 min at 55 °C. Subsequently, a 1:100 suspension of the spores was prepared by mixing the heated sample with sterile natural seawater with agar at 0.8%. From the gelled suspension, 100 µL were distributed in each well and the plate was closed and the borders of the plate sealed with Parafilm®. Subsequently, the plate was placed in wet sediment from the sampling site and incubated for 30 days in the dark, at room temperature. After that period, bacterial growth in the gelled wells was inoculated onto modified M13 medium agar and incubated at 25 °C. Picked, isolated colonies were designated ICT_# (# = well-coordinates). For cryopreservation, isolates were stored in modified M13 medium with 20% (v/v) glycerol and kept at − 80 °C.

### Identification of the strain’s phylogeny

Genomic DNA extraction from isolated colonies of the strains was performed with the E.Z.N.A.® Bacterial DNA Isolation Kit (Omega) according to the manufacturer’s specifications. The 16S rRNA gene was amplified from the genomic DNA by polymerase chain reaction (PCR) with the primers 27F and 1492R, following the protocol described by Bondoso et al. (2011). The PCR products were purified using the Illustra™ GFX™ PCR DNA and Gel Band Purification Kit according to the manufacturer’s specifications. The amplified partial 16S rRNA gene was sequenced by Sanger sequencing at Eurofins Genomics and the sequences were analysed using Geneious R11. The phylogeny was inferred using the 16S-based ID tool in the EzBioCloud platform (Yoon et al. 2017). The consensus 16S rRNA gene sequences obtained were deposited in the National Center for Biotechnology Information Search (NCBI) database. The 16S rRNA gene sequences were also used to generate a phylogenetic tree. The sequences were aligned using CLUSTAL omega together with sequences from the type strains and a *Plantomycetota* strain (*Stieleria sedimenti* ICT_E10.1^ T^, GenBank accession number OL684514) as outgroup. A phylogenetic tree was computed using MEGA X (Kumar et al. 2018) with the Maximum Likelihood method and 1000 bootstraps replicates, the General Time Reversible model (Nei and Kumar 2000) and the Gamma distributed with Invariant Sites (G + I) option activated.

### Strain fermentation and extraction

Strain fermentation and extraction were performed as described by Santos et al. (2022). Briefly, the isolated *Actinomycetota* were fermented in plates containing 25 mL of modified M13 medium (Lage and Bondoso 2011) for 15 days, at 25 °C, in the dark. The cultures were collected and steeped in 100 mL of ethyl-acetate overnight for bioactive molecule extraction. The suspension in ethyl-acetate was collected and the agar was washed twice with 10 mL ethyl-acetate which was added to the extraction suspension. The ethyl-acetate was dried to completeness and the extract was dissolved in 500 µL of 20% (v/v) DMSO. Additionally, an unfermented medium contained in a petri dish was extracted with the same protocol to serve as medium control.

### Antibacterial screening

Antibacterial screening of the extracts was performed against *Escherichia coli* ATCC 25922 and *Staphylococcus aureus* ATCC 29213 as described previously by Santos et al. (2022). In short, single colonies of the target microorganisms were incubated in Nutrient broth (NB) overnight, at 37 °C and 220 rpm. Thereafter, overnight cultures were diluted to obtain an inoculum with 5.0 × 10^5^ cells/mL followed by 90 µL/well of the corresponding diluted inoculum being mixed with 10 µL of extract in triplicate. Streptomycin and ampicillin at 10 mg/mL were used as positive controls for *E. coli* ATCC 25922 and *S. aureus* ATCC 29213, respectively. Negative controls were the solvent (DMSO) and bacterial growth controls. Moreover, medium controls were also added. Absorbance (at 600 nm) was measured in a Thermo Scientific™ Multiskan™ GO. The percentage of growth inhibition was calculated using the following equation:

$$\mathrm{\%inhibition}=100-100\times \frac{({T}_{{\text{F}}E}-{T}_{0E})-({T}_{{\text{F}}B}-{T}_{0B})}{({T}_{{\text{F}}G}-{T}_{0G})-({T}_{{\text{F}}B}-{T}_{0B})}$$

where *T*_0_ is the absorbance at 0 h, *T*_F_ is the absorbance at 24 h, *E* is the extract well, *B* is blank wells and *G* is the control growth wells. Assays were performed three times on different days with a new inocula (*n* = 3). Extracts were considered to have an inhibitory effect only if the target growth was reduced by no less than 50% in at least two assays and the average of all three assays was also above the 50% threshold.

### Antifungal screening

Antifungal screening of the extracts was performed against a variety of pathogenic fungi, including *Candida albicans* ATCC 10231, *Aspergillus fumigatus* ATCC 46645 and *Trichophyton rubrum* FF5, reference strains that belong to the Mycological Laboratory of the Faculty of Pharmacy, University of Porto (Portugal). The antifungal assays were performed in 96-well plates following protocols described by Erbiai et al. (2021) and Benoutman et al. (2022). These follow the Clinical and Laboratory Standard Institute-CLSI guidelines (M38-A2 for filamentous fungi and M27-A3 for yeasts). Succinctly, standardised cultures of each fungus were prepared in medium RPMI-1640: 1.0–3.0 × 10^3^ CFU/mL for *T. rubrum*, 0.4–5.0 × 10^4^ CFU/ml for *A. fumigatus* and 1.0–5.0 × 10^3^ CFU/ml for *C. albicans*. Each well consisted of 180 µL of fungal preparation and 20 µL of culture extract or of positive control. Positive controls consist of 1 µg/mL voriconazole for *A. fumigatus* and 64 µg/mL *fluconazole* for *T. rubrum* and *C. albicans*. Other internal plates consisted of the negative control (180 µL target inoculum + 20 µL of RPMI-1640), solvent control (180 µL target inoculum + 20 µL of 20% (v/v) DMSO) and the blank control (200 µL of RPMI-1640). Growth of the targets was visually inspected after 2 days at 37 °C for *A. fumigatus* and *C. albicans*. For *T. rubrum*, inspection occurred after 7 days at 26 °C. As with the bacterial screenings, antifungal screenings were done in 3 biologically independent assays (*n* = 3).

### LC/MS dereplication of extracts

The dereplication of bioactive extracts was performed in an Agilent 1200 Rapid Resolution HPLC interfaced with a Bruker maXis mass spectrometer. A Zorbax SB-C8 column (2.1 × 30 mm, 3.5 mm particle size) was used, with two solvents used for the mobile phase, both composed of water and acetonitrile with 13 mM ammonium formate and 0.01% trifluoracetic acid. Solvent A was in a ratio of 90:10 water and acetonitrile and solvent B in a 10:90 ratio. The mass spectrometer was operated in positive ESI mode. The putative component identification was obtained by the comparison of UV/vis spectra, retention time and exact mass of the components to the Fundación MEDINA’s database. For components with no matches in MEDINA’s database, the predicted molecular formula and exact mass were searched for in the Chapman and Hall Dictionary of Natural Products (DNP) database. In the event of a possible match being found, considering the exact mass and/or the molecular formula, the producing microorganism and the target assay, the molecule was reported as a suggested component of the fraction (Perez-Victoria et al. 2016).

## Results

### Isolation and identification of strains

**Fig. 1:**
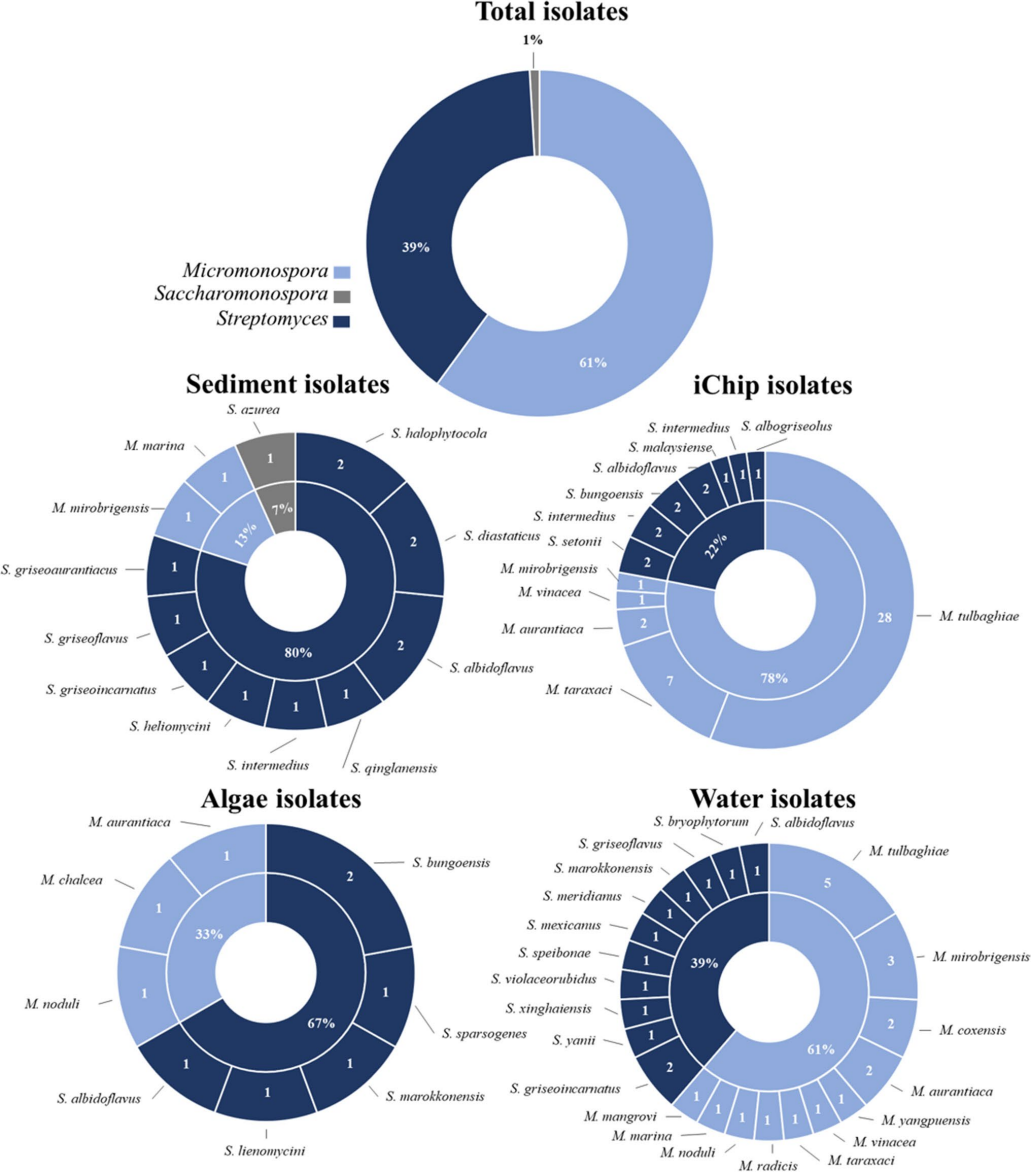
Distribution of isolated strains by genus in total and by each sample origin

Overall, in both the culture-based methods, 105 sporogenous actinomycetotal isolates were obtained from brackish water, brackish sediments and *Ulva* sp. samples. Based on the analysis of the 16S rRNA gene, most of the isolates belong to the genus *Micromonospora* (60% of the total isolates) (Fig. 1), with the remaining strains belonging mostly to the genus *Streptomyces* (39.05% of the total isolates) and one isolate (0.95% of the isolates) to the genus *Saccharomonospora* (Fig. 1). The isolated *Micromonospora* strains (*n* = 63) specified in Table S1, belong to 12 species, the most abundant one was *Micromonospora tulbaghiae* (*n* = 33). This is followed by *Micromonospora taraxaci* (*n* = 8), *M. aurantiaca* and *M. mirobrigensis* (both with *n* = 5). The remaining *Micromonospora* species were represented by one or two isolates (Table S1). Regarding the genus *Streptomyces*, higher diversity was obtained, with 24 different species isolated (Table S1). The most abundant were *S. albidoflavus* (*n* = 6), *S. bungoensis* (*n* = 4) and *S. griseoincarnatus* and *S. intermedius* with three isolates each. Five species were represented by 2 isolates and fourteen species by a single isolate (Table S1). Additionally, strain MTZ3.1^ T^, with 98.62% similarity to *Streptomyces alkaliterrae* OF1^T^, which is below the species threshold of 98.70% (Yarza et al. 2014), represents a novel taxon which was proposed as the type strain of a novel species, *Streptomyces meridianus* (Santos et al. 2023). Considering the different isolation methodologies, 55 strains were isolated from all three sample types using the conventional isolation technique, while with the iChip, which was only applied to the brackish sediments, 50 strains were obtained. By conventionally isolated methodologies, strains belonging to the genus *Streptomyces* (*n* = 30) made up the majority of isolated genera, with the rest belonging to *Micromonospora* (*n* = 24) and *Saccharomonospora* (*n* = 1) (Table S1). The iChip isolates belong to *Micromonospora* (*n* = 39 strains) and *Streptomyces* (*n* = 11 strains) (Table S1). The *Micromonospora* genus is particularly represented by *M. tulbaghiae* for which, using only the iChip, 28 isolates were obtained (Fig. 1). Regarding sample sources from the brackish sediments, 75 strains were isolated, belonging to the genus *Streptomyces* (*n* = 23), *Micromonospora* (*n* = 41) and *Saccharomonospora* (*n* = 1) (Table S1). Between the two different techniques of isolation from sediments, only a few were found to be shared between them. From *Ulva sp*., 9 isolates were obtained that belong to the genus *Streptomyces* (*n* = 6) and *Micromonospora* (*n* = 3), and from the brackish water sample, 31 strains were isolated that belong to the genus *Streptomyces* (*n* = 12) and *Micromonospora* (*n* = 19). A much higher diversity was obtained from the water sample. The overall obtained diversity can be observed in the 16S rRNA gene phylogeny tree (Fig. S1). It is clear the separation between *Micromonospora*, *Streptomyces* and *Saccharomonospora* and that several isolates should be clones of the same microorganism (Fig. S1). The strains 16S rRNA gene sequences were deposited in the NCBI’s GenBank database with accession numbers of MZ475064 for strain MTZ3.1 and OQ326934—OQ327037 for all the others (Table S1).

### Antimicrobial screening and dereplication

Extracts from all strains, except from the *S. bryophytorum* strain MTZ3.30 which lost viability shortly after identification, were tested for antibacterial (*S. aureus* and *E. coli*) and antifungal (*A. fumigatus*, *C. albicans* and *T. rubrum*) activities. Of the 104 strains tested, 26 showed bioactivities against at least one of the pathogens tested. Anti-*S. aureus* activity was present in 12 strains’ extracts (Table 1). The extract of the *M*. *yangpuensis* strain MTZ3.29a induced total growth inhibition and those of the following strains induced almost total inhibition: *S. qinglanensis* strain MTZ1.10, *S. lienomycini* strain MTZ2.3, *S. bungoensis* strain MTZ2.5, *S. bungoensis* strain MTZ3.15 and *M. coxensis* strain MTZ3.16. As for anti-*E. coli* activity, only *M*. *yangpuensis* strain MTZ3.29a and *S. sparsogenes* strain MTZ2.6 extracts induced total and partial inhibition, respectively (Table 1). Moreover, the extracts from these two strains also displayed bioactivity against *S. aureus*. Regarding anti-*A. fumigatus* activity, 3 strains, *S. qinglanensis* strain MTZ1.10, *S. albidoflavus* strain ICT_A4.1a and *S. albogriseolus* strain ICT_A2.1, induced total inhibition. *S. qinglanensis* strain MTZ1.10 and *S. albogriseolus* strain ICT_A4.1a induced total growth inhibition in *C. albicans* and *S. diastaticus* strain MTZ1.4 induced partial growth inhibition of this yeast (Table 1). Anti-*T. rubrum* activity was observed in extracts of 12 strains, with 10 inducing total growth inhibition (*S. sparsogenes* strain MTZ2.6, *S. intermedius* strain ICT_C11.1a and *M. tulbaghiae* strains ICT_B8.1, ICT_C9.2, ICT_C9.4, ICT_D11.2, ICT_D3.3, ICT_D6.1, ICT_D7.1 and MTZ3.13). Partial inhibition was induced by *M. tulbaghiae* strains ICT_A11.3 and ICT_D11.1 (Table 1).

**Table 1:** Average inhibition and putatively identified bioactive compounds and non*-*identified molecules present in the extracts of bioactive strains

Strain ID	Affiliation	Activity					Dereplication	
		*S. aureus*	*E*. *coli*	*A. fumigatus*	*C. albicans*	*T. rubrum*	Identified	Non-identified
MTZ3.16	*Micromonospora coxensis* DSM 45161	79.29	−	−	−	−	*N-*Acetyltyramine, Cyclo(Leu-Pro), Cyclo(Phe-Pro), Cyclo(Phe-Val), Cyclo(Phe-Lxx)	C_17_H_34_O_4_, C_25_H_50_N_10_
MTZ3.15	*Micromonospora coxensis* DSM 45161	83.99	−	−	−	−	Cyclo(Pro-Val), *N-*Acetyltyramine, Cyclo(Leu-Pro), Cyclo(Phe-Lxx)	C_10_H_9_NO_2_, C_12_H_10_N_4_O_2_, C_24_H_41_N_3_O_7_, C_17_H_34_O_4_, C_25_H_50_N_10_
ICT_A11.3	*Micromonospora tulbaghiae* DSM 45142	−	−	−	−	+ / −	*N-*Acetyltyramine, Cyclo(Leu-Pro), Cyclo(Phe-Pro), Cyclo(Phe-Val), Cyclo(Lxx-Lxx), 12-Hydroxy-8,10-octadecadienoic acid	C_8_H_13_NO_3_, C_11_H_15_NO_2_, C_11_H_16_O_3_, C_21_H_33_N_3_O_3_, C_25_H_50_N_10_
ICT_B8.1	*Micromonospora tulbaghiae* DSM 45142	−	−	−	−	+	*N-*Acetyltyramine, Cyclo(Leu-Pro), Cyclo(Phe-Pro), Cyclo(Phe-Val), 12-Hydroxy-8,10-octadecadienoic acid	C_8_H_13_NO_3_, C_11_H_15_NO_2_, C_11_H_16_O_3_, C_20_H_22_O_6_, C_15_H_24_O_3_, C_19_H_32_O_3_S, C_25_H_50_N_10_
ICT_C9.2	*Micromonospora tulbaghiae* DSM 45142	−	−	−	−	+	*N-*Acetyltyramine, Cyclo(Leu-Pro), Cyclo(Phe-Pro), Cyclo(Phe-Val), *N-*Myristylamidopropyl-N,*N-*dimethylbetaine	C_10_H_9_NO_2_, C_8_H_13_NO_3_, C_11_H_15_NO_2_, C_13_H_23_NO_2_, C_21_H_33_N_3_O_3_, C_19_H_32_O_3_S
ICT_C9.4	*Micromonospora tulbaghiae* DSM 45142	−	−	−	−	+	*N-*Acetyltyramine, Cyclo(Leu-Pro), *N-*(2-Phenylethyl)acetamide	C_10_H_9_NO, C_8_H_13_NO_3_, C_11_H_15_NO_2_, C_11_H_16_O_3_, C_10_H_9_NO_2_
ICT_D11.1	*Micromonospora tulbaghiae* DSM 45142	−	−	−	−	+ / −	*N-*Acetyltyramine, Cyclo(Leu-Pro), Cyclo(Phe-Pro)	C_8_H_13_NO_3_, C_11_H_15_NO_2_, C_11_H_16_O_3_, C_10_H_9_NO_2_, C_10_H_13_NO, C_21_H_33_N_3_O_3_, C_19_H_32_O_3_S, C_25_H_50_N_10_
ICT_D11.2	*Micromonospora tulbaghiae* DSM 45142	−	−	−	−	+	*N-*Acetyltyramine, Cyclo(Leu-Pro), Cyclo(Phe-Pro), Cyclo(Phe-Val)	C_8_H_13_NO_3_, C_11_H_15_NO_2_, C_11_H_16_O_3_, C_14_H_31_NO, C_19_H_32_O_3_S, C_25_H_50_N_10_
ICT_D3.3	*Micromonospora tulbaghiae* DSM 45142	−	−	−	−	+	*N-*Acetyltyramine, Cyclo(Leu-Pro), Cyclo(Phe-Pro), Cyclo(Phe-Val)	C_8_H_13_NO_3_, C_11_H_15_NO_2_, C_13_H_14_N_2_O_3_, C_11_H_16_O_3_, C_10_H_9_NO_2_, C_14_H_31_NO, C_25_H_50_N_10_
ICT_D6.1	*Micromonospora tulbaghiae* DSM 45142	−	−	−	−	+	Cyclo(Pro-Val), *N-*Acetyltyramine, Cyclo(Leu-Pro), Cyclo(Phe-Pro), Cyclo(Pro-Trp), Cyclo(Phe-Val), Germicidin G	C_8_H_13_NO_3_, C_11_H_15_NO_2_, C_11_H_16_O_3_, C_19_H_32_O_3_S, C_25_H_50_N_10_
ICT_D7.1	*Micromonospora tulbaghiae* DSM 45142	−	−	−	−	+	*N-*Acetyltyramine, Cyclo(Leu-Pro), Cyclo(Phe-Pro), Cyclo(Lxx-Lxx)	C_8_H_13_NO_3_, C_11_H_15_NO_2_, C_10_H_9_NO_2_, C_10_H_13_NO, C_26_H_42_N_4_O_6_, C_26_H_42_O_5_
MTZ3.13	*Micromonospora tulbaghiae* DSM 45142	−	−	−	−	+	*N-*Acetyltyramine, Cyclo(Leu-Pro), Cyclo(Phe-Pro), Cyclo(Pro-Trp), Cyclo(Phe-Val), Cyclo(Phe-Lxx)	C_8_H_13_NO_3_, C_11_H_15_NO_2_, C_10_H_9_NO_2_
ICT_D9.2	*Micromonospora vinacea* GUI63	64.53	−	−	−	−	Cyclo(Pro-Trp), *N-*Acetyltyramine, Cyclo(Leu-Pro), Cyclo(Phe-Pro), Cyclo(Phe-Val)	C_8_H_13_NO_3_, C_11_H_15_NO_2_, C_11_H_16_O_3_, C_10_H_9_NO_2_, C_13_H_22_O_3_, C_12_H_11_NO_2_S, C_21_H_33_N_3_O_3_, C_20_H_34_N_2_O_6_, C_25_H_50_N_10_
MTZ3.29a	*Micromonospora yangpuensis* DSM 45577	100.0	100.0	−	−	−	*N-*Acetyltyramine, Cyclo(Leu-Pro), Cyclo(Phe-Pro), Cyclo(Phe-Lxx), 12-Hydroxy-8,10-octadecadienoic acid	C_8_H_13_NO_3_, C_11_H_16_O_3_, C_10_H_9_NO_2_, C_18_H_32_O_4_, C_25_H_50_N_10_
ICT_A4.1a	*Streptomyces albidoflavus* DSM 40455	−	−	+	+	−	*N–N-*Dimethyladenosine, *N-*Acetyltyramine, Cyclo(Leu-Pro), Cyclo(Phe-Pro), Germicidin G, Deisovalerylblastmycin, Surugamide A, Surugamide E, Antimycin A11	C_10_H_19_NO_5_, C_8_H_13_NO_3_, C_13_H_22_O_4_, C_12_H_20_O_3_, C_49_H_81_N_9_O_9_, C_25_H_50_N_10_
MTZ2.1	*Streptomyces albidoflavus* DSM 40455	52.29	−	−	−	−	*N-*Acetyltyramine, Cyclo(Leu-Pro), Cyclo(Phe-Pro), Virginiamycin M1, Virginiamycin M2, 12-Hydroxy-8,10-octadecadienoic acid	C_10_H_19_NO_5_, C_11_H_11_NO_3_, C_8_H_13_NO_3_, C_11_H_15_NO_2_, C_13_H_14_N_2_O_3_, C_9_H_12_N_2_O_2_S, putative novel virginiamycin C_28_H_37_N_3_O_6_
ICT_A2.1	*Streptomyces albogriseolus* NRRL B-1305	−	−	+	−	−	*N-*Acetyltyramine, Cyclo(Leu-Pro), Cyclo(Phe-Pro), Cyclo(Phe-Lxx), Germicidin G, Surugamide A, Surugamide E, Antimycin A11, Antimycin A13	C_11_H_11_NO_3_, C_8_H_13_NO_3_, C_13_H_14_N_2_O_3_, C_11_H_16_O_3_, C_12_H_20_O_3_, C_13_H_20_O_2_, C_21_H_30_O_5_, C_25_H_50_N_10_
MTZ2.5	*Streptomyces bungoensis* DSM 41781	80.97	−	−	−	−	Cyclo(Leu-Pro), Cyclo(Phe-Pro), Cyclo(Phe-Val), Limazepine B1/B2, Limazepine A, Germicidin A	C_10_H_16_N_2_O_2_, C_8_H_13_NO_3_, C_15_H_20_N_2_O_4_, C_15_H_18_O_6_, C_12_H_16_N_2_O_2_, C_14_H_17_NO_2_, C_20_H_22_O_6_, C_12_H_18_O_3_, C_34_H_42_N_4_O_7_, C_18_H_32_O_4_, C_19_H_32_O_3_S, C_25_H_50_N_10_
MTZ1.4	*Streptomyces diastaticus* NBRC 3714	−	−	−	+ / −	−	*N-*Acetyltyramine, Cyclo(Leu-Pro), Cyclo(Phe-Pro), Germicidin G, Alteramide A, Dihydromaltophilin, Surugamide A, Surugamide E, 12-Hydroxy-8,10-octadecadienoic acid	C_10_H_16_N_2_O_2_, C_8_H_13_NO_3_, C_11_H_11_NO_3_, C_11_H_16_O_3_, C_13_H_22_O_4_, C_18_H_27_NO_5_, C_12_H_20_O_3_, C_13_H_20_O_2_, C_49_H_81_N_9_O_9_, C_18_H_32_O_4_, C_25_H_50_N_10_
MTZ1.18	*Streptomyces halophytocola* KLBMP 1284	60.47	−	−	−	−	Cyclo(Pro-Val), *N-*Acetyltyramine, Cyclo(Leu-Pro), Cyclo(Phe-Pro)	C_8_H_13_NO_3_, C_8_H_8_N_2_S, C_10_H_10_O_4_, C_11_H_16_O_3_, C_11_H_14_O_4_, C_12_H_11_NO_2_S, C_12_H_18_O_5_, C_18_H_30_O_3_, C_18_H_30_O_2_, C_25_H_44_O_3_S_2_
MTZ1.5	*Streptomyces heliomycini* NBRC 15899	52.36	−	−	−	−	*N-*Acetyltyramine, Cyclo(Leu-Pro), Cyclo(Phe-Pro)	C_8_H_13_NO_3_, C_12_H_20_O_3_, C_13_H_22_O_3_, C_22_H_30_O_5_, C_12_H_22_O_3_, C_16_H_34_O_6_S, C_18_H_32_O_4_, C_19_H_32_O_3_S, C_22_H_16_O_6_, C_25_H_50_N_10_
ICT_C11.1a	*Streptomyces intermedius* NBRC 13049	−	−	−	−	+	*N–N-*Dimethyladenosine, *N-*Acetyltyramine, Cyclo(Leu-Pro), IAA, Cyclo(Phe-Pro), Germicidin G	C_10_H_19_NO_5_, C_8_H_13_NO_3_, C_10_H_11_NO_4_, C_13_H_22_O_4_, C_11_H_16_O_3_, C_12_H_20_O_3_, C_13_H_20_O_2_ related to germicidin, C_18_H_30_O_3_, C_25_H_50_N_10_
MTZ2.3	*Streptomyces lienomycini* LMG 20091	78.46	−	−	−	−	*N–N-*Dimethyladenosine, *N-*Acetyltyramine, Cyclo(Leu-Pro), IAA, Cyclo(Phe-Pro), Germicidin A	C_10_H_19_NO_5_, C_25_H_49_N_5_O, C_12_H_14_O_4_, C_18_H_32_O_4_, C_25_H_50_N_10_
MTZ1.10	*Streptomyces qinglanensis* 172,205	78.20	−	+	+	−	Cyclo(Leu-Pro), Cyclo(Phe-Pro), Cyclo(Phe-Val), 12-Hydroxy-8,10-octadecadienoic acid	C_10_H_19_NO_5_, C_7_H_11_NO_3_, C_8_H_13_NO_3_, C_11_H_15_NO_2_, C_13_H_15_N_2_O_3_, C_15_H_18_O_6_, C_15_H_16_O_5_, C_18_H_16_N_2_, C_18_H_32_O_4_, C_21_H_30_O_5_, C_25_H_50_N_10_
MTZ2.6	*Streptomyces sparsogenes* ATCC 25498	64.54	51.68	−	−	+	Tubercidin, *N-*Acetyltyramine, Cyclo(Leu-Pro), Cyclo(Phe-Pro), Antibiotic X 14952B	C_10_H_19_NO_5_, C_8_H_13_NO_3_, C_12_H_12_N_2_O_2_, C_11_H_16_O_3_, C_10_H_11_NO_3_, C_25_H_50_N_10_
MTZ3.20	*Streptomyces speibonae* NRRL B-24240	53.13	−	−	−	−	Cyclo(Pro-Val), *N-*Acetyltyramine, Cyclo(Leu-Pro), Cyclo(Phe-Pro), Cyclo(Phe-Val), Cyclo(Phe-Lxx), 12-Hydroxy-8,10-octadecadienoic acid	C_10_H_16_NO_5_, C_10_H_9_NO, C_8_H_5_NO_3_, C_9_H_12_N_2_O_2_S, C_11_H_16_O_3_, C_13_H_20_O_2_, C_18_H_32_O_4_, C_25_H_50_N_10_

+ , total growth inhibition; + / − , partial growth inhibition; − , no growth inhibition

All the bioactive extracts were analysed by HPLC/HRMS. Dereplication is a critical step in natural product discovery, as it putatively detects and identifies known molecules associated to the observed biological activity in this early stage of the screening process, which is important to avoid rediscovery. The results obtained are presented in Table 1, showing several known natural products. These are frequently detected in the extracts during dereplication, which is the case of diketopiperazines such as Cyclo(Leu-Pro), Cyclo(Phe-Pro), Cyclo(Phe-Val), Cyclo(Phe-Lxx) and *N*-acetyltyramine (Santos et al. 2022) (Table 1). Other known molecules with no relevant bioactivity identified include the auxin indole-3-acetic acid (IAA), the 12-Hydroxy-8,10-octadecadienoic acid, the *N*–*N*-Dimethyladenosine, the *N*-Myristylamidopropyl-*N*,*N*-Dimethylbetaine and the *N*-(2-Phenylethyl)acetamide (Table 1). However, several relevant bioactive molecules were also found. Alteramide A is a macrocyclic lactam antibiotic with antifungal and cytotoxic properties (Shigemori et al. 1992). Antibiotic X 14952B is a homologue of the macrolide antifungal Venturicidin B with antibacterial activity (Omura et al. 1985). Antimycins are macrolide antibiotic complexes, with antibacterial and antifungal properties and high toxicity to animals (Watanabe et al. 1957; Rieske 1967; Ishiyama et al. 1976; Hosotani et al. 2005; Yan et al. 2010). Antimycin A11, Antimycin A13 and Deisovalerylblastmycin were putatively identified. Dihydromaltophilin is, like alteramide A, a macrolactam-tetramic acid antibiotic with several bioactive properties, including antifungal and cytotoxic effects (Graupner et al. 1997). Germicidins A and G are pyranones spore regulators for sporogenous *Actinomycetota* (Petersen et al. 1993; Xu et al. 2011). Limazepine A and B1/B2 (which are epimers and impossible to distinguish with only HPLC/HRMS) were also detected. Limazepines are pyrrolo[1,4]benzodiazepines with antibacterial activity isolated from *Streptomyces* sp. ICBB 8177 (Fotso et al. 2009). Surugamide A and E are cyclic octapeptides with cytotoxic properties (Takada et al. 2013). Virginiamycins are a family of antibiotics which are usually used in combination with type A (polyunsaturated macrolactones) and type B (peptidic macrolactones), which in conjunction, give them wide antibacterial effects (Kingston et al. 1983; Schlessinger and Li 1996). Virginiamycin M1, which is a polyunsaturated macrolactone, and Virginiamycin M2, a peptidic macrolactone, were observed in the dereplication. Tubercidin, which is a macrolide antibiotic with antibacterial, antifungal, antiviral and cytotoxic properties (Mizuno et al. 1963), was also observed (Table 1). Several components remained unidentified, as no match was obtained in either MEDINA’s or the DNP databases. In total, 62 molecules were not fully identified. Of the non-identified molecules, 18 had molecular formulae that did not match any in the DNP (Table S2), 9 had one correspondence (Table S2), 11 had two correspondences (Table S2), 7 had three correspondences (Table S2), 7 had four correspondences (Table S2), 3 had 5 correspondences (Table S2), two had 6 correspondences (Table S2), one had seven correspondences (Table S2), one had eight correspondences (Table S2), two had ten correspondences (Table S2) and one had twelve correspondences (Table S2). Some of these known molecules have relevant bioactivities, such as Yanglingmycin and Madurastatin B3, which exhibit broad spectrum antibacterial activity or Fugomycin, 2-Butyl-5-propylresorcinol and Xenocyloin A that possess antibacterial and antifungal properties. Furthermore, some of the non-identified molecules are present in several extracts. These are compound with a molecular formula (MF) of C_8_H_13_NO_3_ appearing in 22 extracts, C_25_H_50_N_10_ appears in 20 extracts, C_11_H_16_O_3_ in 15; C_11_H_15_NO_2_ in 13; C_10_H_9_NO_2_ in 9; C_19_H_32_O_3_S and C_18_H_32_O_4_ in 7; C_10_H_19_NO_5_ in 6; C_12_H_20_O_3_ in 5; C_21_H_33_N_3_O_3_ and C_13_H_20_O_2_ in 4; C_13_H_14_N_2_O_3_ C_13_H_22_O_4_ and C_11_H_11_NO_3_ in 3; C_17_H_34_O_4_, C_20_H_22_O_6_, C_10_H_13_NO, C_14_H_31_NO, C_13_H_22_O_3_, C_12_H_11_NO_2_S, C_9_H_12_N_2_O_2_S, C_21_H_30_O_5_, C_10_H_16_N_2_O_2_, C_15_H_18_O_6_, C_18_H_30_O_3_ and C_49_H_81_N_9_O_9_ in 2 extracts; and 36 additional molecules in different unique extracts. One of these may be a putatively novel virginiamycin, with a MF of C_28_H_37_N_3_O_6_. The MF C_13_H_20_O_2_ also appears to be related to Germicidin (Table 1).

## Discussion

Due to their relevant ecological, social, cultural, and economic value, estuaries exert a substantial influence on humans and their well-being. Estuaries are important habitats for many species of birds, fish, and invertebrates and, as they are biologically productive areas, harbour a rich microbial life. These make estuaries prime candidates for microbiological studies and in the search of biotechnologically useful bacteria. Their communities are usually dominated by *Pseudomonadota*, but other phyla such as *Cyanobacteria*, *Bacteroidota*, *Actinomycetota* and *Planctomycetota* are present therein (Yi et al. 2020; Wang et al. 2021; Vitorino et al. 2022). The Tagus River estuary, which includes “Tagus Estuary Natural Reserve”, is ecologically very important for wildlife and a hotspot for microbial organisms. In fact, very high numbers of bacterial cells in its water and sediment have been referred to (Santos et al. 2007).

In this study with its focus on sporogenous *Actinomycetota*, in total, 105 strains were isolated from the 3 kinds of samples from the Tagus River estuary. More *Actinomycetota* strains were recovered from sediments (conventional isolation method and the iChip), a total of 65 (62%), being 24 different taxa from 3 genera. The microorganisms present in the water sample were also diverse, as 31 strains (29%) belong to 21 different taxa, including a novel taxon, strain MTZ3.1^ T^ *Streptomyces meridianus*. From *Ulva* sp., 9 isolates from 8 different taxa were obtained (9%). *S. albidoflavus*, a species inhabiting diverse habitats (Cheng et al. 2015), was the only strain isolated from the 4 combinations of samples/methodologies. Taking a closer look at the methodologies used for isolation, the iChip methodology allowed the isolation of 50 strains from 12 different taxa, and from sediments with the conventional isolation technique, only 15 strains from 12 different taxa were obtained, with *M. mirobrigensis*, *S. albidoflavus* and *S. intermedius* being the only common isolated taxa. A noteworthy fact is that in the iChip methodology, a very high number of isolated strains belong to *M. tulbaghiae* (28 strains). This taxon, originally isolated from leaves of the indigenous South African plant *Tulbaghia violacea* (Kirby and Meyers 2010), was also isolated in our study from the water sample. Similarly, *M. taraxaci* and *M. vinacea* were only isolated using iChip in sediments and from the water column. Besides *Streptomyces* and *Micromonospora*, in our previous study using the iChip approach (Santos et al. 2022), *Nocardiopsis* strains were also isolated. A study of the Yalu River estuary, in Northern China showed that, in sediment samples from 15 sites, a total of 173 strains were isolated. These strains belonged to 13 genera, with about 71% of the isolates from the genus *Streptomyces* and only 10% to *Micromonospora* (Yu et al. 2015). In another study in Southern China, in the “Beilun Estuary National Nature Reserve”, from plant tissue samples from mangrove trees, 101 strains related to the phylum *Actinomycetota* were isolated. Of these, 33% belonged to the genus *Streptomyces* and 2% to *Micromonospora* (Jiang et al. 2018). In both studies, a majority of the isolated sporogenous *Actinomycetota* are *Streptomyces*. However, in a study with the same aim to specifically obtain sporogenous *Actinomycetota* from marine sediments in Cepães beach, Esposende, Portugal, Ribeiro et al. (2020) obtained 52 *Actinomycetota* isolates, of which, 67% belonged to the genus *Micromonospora*, 17% to *Streptomyces*, 8% to *Arthrobacter* and 2% to *Nocardiopsis*, *Herbiconiux*, *Polymorphospora* and *Actinomadura*. Percentage-wise, these last results align with those obtained in our study, where the majority (60%) were *Micromonospora* and 39% to *Streptomyces*. It has been hypothesised that *Streptomyces* are the dominant *Actinomycetota* in terrestrial sources, which may contribute to microbial community composition in estuarine environments (Jensen et al. 1991; Takizawa et al. 1993; Bredholdt et al. 2007; Terahara et al. 2013). *Micromonospora* is widely distributed but commonly found in marine environments (Hirsch and Valdés 2010). Moreover, Terahara et al. (2013) showed that *Micromonospora* spores are more heat-resistant than the spores from *Streptomyces*.

Based on the obtained results, the two methodologies applied to sediments showed to be complementary as they allowed the obtainment of a broader diversity. Additionally, the present study shows the great diversity of both *Micromonospora* and *Streptomyces* species present in the river Tagus estuary.

Regarding the bioactivity screening of the 104 strains tested, extracts from 26 strains displayed bioactivity against one of the targets, mainly against *S. aureus* ATCC 29213 and *T. rubrum* FF5, both with 12 bioactive extracts. As for the other targets, only 3 extracts were bioactive against *A. fumigatus* ATCC 240305 and *C. albicans* ATCC 10231 and 2 against *E. coli* ATCC 25922. Particularly interesting and promising activities are seen in the extracts of 18 strains that had activities higher than 50% against *S. aureus* ATCC 29213 and *E. coli* ATCC 25922 or induced reduction of fungal growth (Table 1). The extracts of *M. yangpuensis* MTZ3.29a and *S. sparsogenes* MTZ2.6 were the only bioactive ones against both bacterial targets and *M. yangpuensis* MTZ3.29a’s extract induced total growth inhibition. *S. albidoflavus* ICT_A4.1a and *S. qinglanensis* MTZ1.10 were the only strains that inhibited the growth of the fungi *A. fumigatus* ATCC 240305 and *C. albicans* ATCC 10231. The extracts from 10 strains of *M. tulbaghiae* showed total or partial inhibition of *T. rubrum* FF5 (Table 1).

The genera herein isolated are some of the more relevant ones regarding the discovery of novel and biotechnologically useful natural products (Blunt et al. 2018; Carro et al. 2018; Carroll et al. 2019, 2020, 2023; Santos et al. 2020). Data on the bioactivity of strains from the genus *Micromonospora* are abundant. Molecules like 11-Deoxydoxorubicin (Muindi et al. 1984), Bravomicin A (Banskota et al. 2009), Gentamicin B_1_ (Ni et al. 2016), Micromonomycin (Yang et al. 2004) and Neorustmicins B, C and D (Nakayama et al. 1986) are compounds with antibacterial and antifungal properties isolated from strains belonging to this genus. In fact, the DNP shows 542 natural products assigned to this genus. The species herein isolated show evident biotechnological potential. From *M. coxensis*, two natural products have been isolated, Deoxydehydrochorismic acid and Diacidene, both isolated from a marine-derived *M. coxensis* (Ohlendorf et al. 2012). *M. yangpuensis* is the source of five compounds, Yangpumicins A–E (Yan et al. 2017). Yangpumicin A has potent cytotoxic effects and is structurally similar to the enediyne antibiotic Uncialamycin (Davies et al. 2005; Yan et al. 2017). *M. marina* has 4 molecules reported, 3 terpenoids with no bioactive properties reported (Rinkel and Dickschat 2019) and the depsipeptide with cytotoxic effects and RNA synthesis inhibitor, Thiocoraline (Romero et al. 1997). Forty-two different compounds have been reported from *M. chalcea*, which include Neorustmicins B, C and D (Nakayama et al. 1986). No reported compounds were assigned to *M. aurantiaca*, *M. mangrovi*, *M. mirobrigensis*, *M. noduli*, *M. radicis*, *M. taraxaci*, *M. tulbaghiae* or *M. vinacea*. However, in our study, these strains were able to produce extracts with antimicrobial properties that may be related to the several molecules identified in the extracts (Table 1). Moreover, 26 molecules were putatively detected but not identified (Table 1).

Literature on the genus *Saccharomonospora* shows their capacity to produce biologically relevant natural products. In the DNP, 17 molecules have been reported in this genus, with several showing antimicrobial and cytotoxic properties. For example, Taromycin A and B are lipopeptide antibiotics isolated from a biosynthetic gene cluster from *Saccharomonospora* sp. CNQ-490 expressed on a *Streptomyces coeliecolor*. Both Taromycins have a strong anti-Gram-positive bacteria effect (Reynolds et al. 2018). Saccharonol B is an isocoumarin isolated from *Saccharomonospora azurea* MTCC11714 with cytotoxic properties (Singh et al. 2013). In the conditions herein tested, strains MTZ1.15 (identified as *S. azurea*) did not show a bioactive effect against the microbial targets tested.

The genus *Streptomyces* is widely regarded as the exemplar for natural products and drug discovery research. Just in the DNP, over 9000 molecules are assigned to this genus, many of which are used for or are the basis of several pharmaceutical formulations. Some notable natural products include Streptomycin, the first aminoglycoside antibiotic discovered, Chloramphenicol, which was the first line drug to cure typhoid infections (Dunitz 1952), and Actinomycin D, the first antibiotic with anti-cancer properties (Bullock and Johnson 1957). Regarding the strains isolated in this study, data show their close association with strains already known to be producers of biotechnologically relevant molecules. Data on the DNP shows that *S. albidoflavus* is the producer of four natural products: (1) Antimycin A, which, as discussed earlier, is a macrolide antibiotic complex, which is highly toxic to animals; (2) Fredericamycin A, an antitumour agent (Misra et al. 1982); (3) MKN-003C, an antifouling agent (Cho et al. 2001); and (4) Albaflavenone, a sesquiterpene ketone with activity against *Bacillus subtilis* (Gurtler et al. 1994). According to the DNP, 10 compounds are produced by *S. albogriseolus* species, which includes Amphomycin (Bodanszky et al. 1973), a lipopeptide antibiotic with anti-Gram-positive spectrum, Cephamycin C (Nagarajan et al. 1971), a cephalosporin antibiotic that is selective towards Gram-negative bacteria, and the angucycline antibiotic Vineomycin A_1_ (Omura et al. 1977), with potent antibacterial and cytotoxic activities. *S. diastaticus* has 40 associated natural products, including Longicatenamycin, a chlorinated cyclic peptide with anti-Gram-positive properties (Shoji et al. 1970), and Phenazinolines A–E, diphenazines with antibacterial and cytotoxic characteristics (Ding et al. 2011). The DNP shows that four compounds have been reported from *S. griseoaurantiacus*, including Diperamycin, a depsipeptide antibiotic with anti-Gram-positive activity (Matsumoto et al. 1998), and Griseolic acid A–C, cyclic-AMP phosphodiesterase inhibitors (Iijima et al. 1985). *S. griseoflavus* has 42 reports in the DNP, from which are examples, Aborycin, that is a tryciclic peptide antibiotic that inhibits HIV’s protease and is active against Gram-positive bacteria (Helynck et al. 1993), Adenomycin, a nucleoside antibiotic that has broad-spectrum activity and anticancer properties (Ogita et al. 1980), and Colabomycins A–D, manumycin-type antibiotics with anti-Gram-positive and cytotoxic properties (Grote et al. 1988; Dick et al. 1994). *S. griseoincarnatus* has 7 hits in the DNP, including Tetracenomycin D, an antimicrobial and antitumour agent (Sajid et al. 2011). Peptides with antimicrobial and antitumour activity, Thioholgamides A and B, were isolated from *S. malaysiense* (Kjaerulff et al. 2017). *S. setonii* has 5 entries in the DNP and it is the origin of 16-Deethylindanomycin, an ionophore with anti-Gram-positive bacteria (Larsen et al. 1988). *S. sparsogenes* is the origin of Sparsomycins A, A_1_ and A_2_, antibiotics that inhibit protein biosynthesis in both prokaryotes and eukaryotes (Wiley and MacKellar 1976). *S. xinghaiensis*, with 13 compounds reported in the DNP database, is the source of Xiamycins, pentacyclic indolosesquiterpenes with anti-HIV, antibacterial, antifungal and anticancer properties and Dixiamycins which are N–N-coupled dimeric indolosesquiterpenes (Ding et al. 2010; Zhang et al. 2012). No reported molecules were found for the remaining species. Like the *Micromonospora* strains, the *Streptomyces* strains displayed remarkable bioactivities, some in line with the available literature. This is the case for the presence of Surugamide A, Surugamide E and Antimycin A11 in the ICT_A4.1a *S. albidoflavus* which showed relevant antifungal activity against *A. fumigatus* ATCC 240305 and *C. albicans* ATCC 10231 or the presence of Limazepines A and B1/B2 on MTZ2.5 *S. bungoensis* which had potent activity against *S. aureus* ATCC 29213 (Table 1). Moreover, 49 molecules were putatively detected but not identified (Table 1). Of the 49 molecules detected in *Streptomyces* extracts, 11 molecules (C_17_H_34_O_4_, C_10_H_9_NO, C_8_H_13_NO_3_, C_11_H_15_NO_2_, C_11_H_16_O_3_, C_19_H_32_O_3_S, C_17_H_34_O_4_, C_20_H_22_O_6_, C_12_H_11_NO_2_S, C_13_H_22_O3 and C_25_H_50_N_10_) are also putatively found in the extracts from the bioactive *Micromonospora* strains. However, two molecules appear to be of special interest, C_28_H_37_N_3_O_6_ and C_18_H_30_O_3_. The molecule with the formula C_28_H_37_N_3_O_6_ is a putatively new virginiamycin derivative, while the molecule with the formula C_18_H_30_O_3_ seems to be related to Germicidins. Overall, these results are continued evidence that bacteria from this phylum have the ability to produce bioactive natural products, as the extracts of the strains studied showed antimicrobial activity and putatively contain bioactive compounds, which may be possible novel natural products.

## Conclusions

A high number of sporogenous *Actinomycetota* were recovered from the different samples from the Tagus River estuary, including a putative novel taxon, MTZ3.1^ T^ *Streptomyces meridianus*. The isolated *Actinomycetota* showed strong bioactivity against both Gram-stain-positive and negative targets, in particular against *S. aureus* ATCC 29213, and also against the pathogenic fungi tested, in particular the filamentous *T. rubrum* FF5. The dereplication of the bioactive extracts showed the putative presence of several known bioactive molecules, like Virginiamycins, Antimycins and Surugamides, as well as of some with unknown identity that might constitute new natural products, with antimicrobial activity. Microbiologically, the Tagus River estuary has been revealed to be an interesting site for the isolation of *Actinomycetota*. Furthermore, the bioactivities observed show the usefulness of *Actinomycetota* for the discovery of bioactive natural products.

## Supplementary Information

Below is the link to the electronic supplementary material.

Supplementary file1 (PDF 877 KB)

## Data Availability

The authors state that all data generated or analysed during this study are included in this published article and its supplementary information files.
