# Supplementary material for: Unveiling the bioactive potential of Actinomycetota from the Tagus River estuary
Source: Int Microbiol. 2024 Jan 18;27(5):1357–72. doi: 10.1007/s10123-024-00483-0 (PMC11452475; doi:10.1007/s10123-024-00483-0)
Supplement: Supplementary file 1 — Supplementary file1 (PDF 877 KB) [file 10123_2024_483_MOESM1_ESM.pdf]

Unveiling the bioactive potential of Actinomycetota from the Tagus River estuary.

José Diogo Neves dos Santos[1,2*]; Eugénia Pinto[2,3]; Jesús Martín[4]; Francisca Vicente[4]; Fernando Reyes[4]; Olga Maria Lage[1,2]

[1]Department of Biology, Faculty of Sciences, University of Porto, Rua do Campo Alegre S/N 4169-007 Porto, Portugal

[2]Interdisciplinary Centre of Marine and Environmental Research, University of Porto, Terminal de Cruzeiros do Porto de Leixões, Avenida General Norton de Matos, S/N, 4450-208 Matosinhos, Portugal

[3]Laboratory of Microbiology, Department of Biological Sciences, Faculty of Pharmacy, University of Porto, Rua Jorge de Viterbo Ferreira 228 4050-313 Porto, Portugal

[4]Fundación MEDINA, Centro de Excelencia en Investigación de Medicamentos Innovadores en Andalucía, Avenida del Conocimiento, 34 Parque Tecnológico de Ciencias de la Salud, 18016 Granada, Spain

[*]Correspondence: zesantox@gmail.com, Tel (+351910903938), ORCID: 0000-0002-9693-4857

Table S1 - Isolated strains ID, affiliation and 16S rRNA gene GenBank accession number.

Strain ID	Affiliation<br>%	 Taxonomic unit	GenBank accession number.	Sample Origin	Isolation Technique
ICT_A11.4	100.0	*Micromonospora aurantiaca* ATCC 27029	OQ326991	Brackish Sediment	iChip
ICT_C7.1	100.0	*Micromonospora aurantiaca* ATCC 27029	OQ327002	Brackish Sediment	iChip
MTZ2.8	99.85	*Micromonospora aurantiaca* ATCC 27029	OQ326959	*Ulva* sp.	Conventional
MTZ3.23	100.0	*Micromonospora aurantiaca* ATCC 27029	OQ326950	Brackish water	Conventional
MTZ3.28	100.0	*Micromonospora aurantiaca* ATCC 27029	OQ326953	Brackish water	Conventional
MTZ2.7	99.92	*Micromonospora chalcea* DSM 43026	OQ327025	*Ulva* sp.	Conventional
MTZ3.15	99.77	*Micromonospora coxensis* DSM 45161	OQ326986	Brackish water	Conventional
MTZ3.16	99.53	*Micromonospora coxensis* DSM 45161	OQ327031	Brackish water	Conventional
MTZ3.26	99.37	*Micromonospora mangrovi* 2803GPT1-18	OQ326964	Brackish water	Conventional
MTZ1.12	99.92	*Micromonospora marina* DSM 45555	OQ326975	Brackish Sediment	Conventional
MTZ3.14	99.92	*Micromonospora marina* DSM 45555	OQ326958	Brackish water	Conventional
ICT_B6.3	99.47	*Micromonospora mirobrigensis* DSM 44830	OQ326981	Brackish Sediment	iChip
MTZ1.14	99.84	*Micromonospora mirobrigensis* DSM 44830	OQ326976	Brackish Sediment	Conventional
MTZ3.11	99.85	*Micromonospora mirobrigensis* DSM 44830	OQ326944	Brackish water	Conventional
MTZ3.12	99.84	*Micromonospora mirobrigensis* DSM 44830	OQ326948	Brackish water	Conventional
MTZ3.9	99.85	*Micromonospora mirobrigensis* DSM 44830	OQ326960	Brackish water	Conventional
MTZ2.9	99.83	*Micromonospora noduli* GUI43	OQ326954	*Ulva* sp.	Conventional
MTZ3.24	100.0	*Micromonospora noduli* GUI43	OQ326969	Brackish water	Conventional
MTZ3.27	99.77	*Micromonospora radicis* AZ1-13	OQ326972	Brackish water	Conventional
ICT_B1.2	100.0	*Micromonospora taraxaci* DSM 45885	OQ327017	Brackish Sediment	iChip
ICT_C9.1	99.85	*Micromonospora taraxaci* DSM 45885	OQ327021	Brackish Sediment	iChip
ICT_D11.3	99.84	*Micromonospora taraxaci* DSM 45885	OQ327020	Brackish Sediment	iChip
ICT_D11.3a	99.85	*Micromonospora taraxaci* DSM 45885	OQ327026	Brackish Sediment	iChip
ICT_D11.3b	99.84	*Micromonospora taraxaci* DSM 45885	OQ327027	Brackish Sediment	iChip
ICT_D3.2	99.85	*Micromonospora taraxaci* DSM 45885	OQ327023	Brackish Sediment	iChip
ICT_D6.2	99.84	*Micromonospora taraxaci* DSM 45885	OQ327035	Brackish Sediment	iChip
MTZ3.21	99.85	*Micromonospora taraxaci* DSM 45885	OQ326961	Brackish water	Conventional
ICT_A11.1	100.0	*Micromonospora tulbaghiae* DSM 45142	OQ326987	Brackish Sediment	iChip
ICT_A11.2	100.0	*Micromonospora tulbaghiae* DSM 45142	OQ326980	Brackish Sediment	iChip
ICT_A11.3	100.0	*Micromonospora tulbaghiae* DSM 45142	OQ326994	Brackish Sediment	iChip
ICT_A5.1	100.0	*Micromonospora tulbaghiae* DSM 45142	OQ326995	Brackish Sediment	iChip
ICT_B1.1	100.0	*Micromonospora tulbaghiae* DSM 45142	OQ326990	Brackish Sediment	iChip
ICT_B1.3	100.0	*Micromonospora tulbaghiae* DSM 45142	OQ327037	Brackish Sediment	iChip
ICT_B11.1	100.0	*Micromonospora tulbaghiae* DSM 45142	OQ326988	Brackish Sediment	iChip
ICT_B11.2	100.0	*Micromonospora tulbaghiae* DSM 45142	OQ327015	Brackish Sediment	iChip
ICT_B12.3	100.0	*Micromonospora tulbaghiae* DSM 45142	OQ327022	Brackish Sediment	iChip
ICT_B6.1	100.0	*Micromonospora tulbaghiae* DSM 45142	OQ326993	Brackish Sediment	iChip
ICT_B6.2	100.0	*Micromonospora tulbaghiae* DSM 45142	OQ326978	Brackish Sediment	iChip
ICT_B8.1	100.0	*Micromonospora tulbaghiae* DSM 45142	OQ327001	Brackish Sediment	iChip
ICT_B8.2	100.0	*Micromonospora tulbaghiae* DSM 45142	OQ327000	Brackish Sediment	iChip
ICT_B8.3	100.0	*Micromonospora tulbaghiae* DSM 45142	OQ327032	Brackish Sediment	iChip
ICT_C8.1	100.0	*Micromonospora tulbaghiae* DSM 45142	OQ326999	Brackish Sediment	iChip
ICT_C9.2	100.0	*Micromonospora tulbaghiae* DSM 45142	OQ327003	Brackish Sediment	iChip
ICT_C9.3	100.0	*Micromonospora tulbaghiae* DSM 45142	OQ327010	Brackish Sediment	iChip
ICT_C9.4	100.0	*Micromonospora tulbaghiae* DSM 45142	OQ327009	Brackish Sediment	iChip
ICT_D1.1	100.0	*Micromonospora tulbaghiae* DSM 45142	OQ327013	Brackish Sediment	iChip
ICT_D11.1	100.0	*Micromonospora tulbaghiae* DSM 45142	OQ327011	Brackish Sediment	iChip
ICT_D11.2	100.0	*Micromonospora tulbaghiae* DSM 45142	OQ327019	Brackish Sediment	iChip
ICT_D3.1	100.0	*Micromonospora tulbaghiae* DSM 45142	OQ327018	Brackish Sediment	iChip
ICT_D3.3	100.0	*Micromonospora tulbaghiae* DSM 45142	OQ327014	Brackish Sediment	iChip

ICT_D6.1	100.0	*Micromonospora tulbaghiae* DSM 45142	OQ326997	Brackish Sediment	iChip
ICT_D6.3	100.0	*Micromonospora tulbaghiae* DSM 45142	OQ326998	Brackish Sediment	iChip
ICT_D7.1	100.0	*Micromonospora tulbaghiae* DSM 45142	OQ327006	Brackish Sediment	iChip
ICT_D7.2	100.0	*Micromonospora tulbaghiae* DSM 45142	OQ327005	Brackish Sediment	iChip
ICT_D9.3	100.0	*Micromonospora tulbaghiae* DSM 45142	OQ327012	Brackish Sediment	iChip
MTZ3.10	100.0	*Micromonospora tulbaghiae* DSM 45142	OQ326957	Brackish water	Conventional
MTZ3.13	100.0	*Micromonospora tulbaghiae* DSM 45142	OQ326949	Brackish water	Conventional
MTZ3.18	100.0	*Micromonospora tulbaghiae* DSM 45142	OQ326956	Brackish water	Conventional
MTZ3.22	100.0	*Micromonospora tulbaghiae* DSM 45142	OQ326951	Brackish water	Conventional
MTZ3.8	100.0	*Micromonospora tulbaghiae* DSM 45142	OQ326943	Brackish water	Conventional
ICT_D9.2	99.92	*Micromonospora vinacea* GUI63	OQ327024	Brackish Sediment	iChip
MTZ3.25	100.0	*Micromonospora vinacea* GUI63	OQ326962	Brackish water	Conventional
MTZ3.29a	100.0	*Micromonospora yangpuensis* DSM 45577	OQ326971	Brackish water	Conventional
MTZ1.15	100.0	*Saccharomonospora azurea* NA-128	OQ327008	Brackish Sediment	Conventional
ICT_A4.1a	99.68	*Streptomyces albidoflavus* DSM 40455	OQ326984	Brackish Sediment	iChip
ICT_A4.1b	99.69	*Streptomyces albidoflavus* DSM 40455	OQ326983	Brackish Sediment	iChip
MTZ1.13b	99.70	*Streptomyces albidoflavus* DSM 40455	OQ326966	Brackish Sediment	Conventional
MTZ1.6	99.69	*Streptomyces albidoflavus* DSM 40455	OQ326939	Brackish Sediment	Conventional
MTZ2.1	99.70	*Streptomyces albidoflavus* DSM 40455	OQ326937	*Ulva* sp.	Conventional
MTZ3.6	99.68	*Streptomyces albidoflavus* DSM 40455	OQ326946	Brackish water	Conventional
ICT_A2.1	100.0	*Streptomyces albogriseolus* NRRL B-1305	OQ326996	Brackish Sediment	iChip
MTZ3.30	99.83	*Streptomyces bryophytorum* NEAU-HZ10	OQ327030	Brackish water	Conventional
ICT_D4.1a	99.68	*Streptomyces bungoensis* DSM 41781	OQ327028	Brackish Sediment	iChip
ICT_D4.1b	99.69	*Streptomyces bungoensis* DSM 41781	OQ327029	Brackish Sediment	iChip
MTZ2.2	99.69	*Streptomyces bungoensis* DSM 41781	OQ326938	*Ulva* sp.	Conventional
MTZ2.5	99.91	*Streptomyces bungoensis* DSM 41781	OQ326947	*Ulva* sp.	Conventional
MTZ1.4	100.0	*Streptomyces diastaticus* NBRC 3714	OQ327007	Brackish Sediment	Conventional
MTZ1.7	100.0	*Streptomyces diastaticus* NBRC 3714	OQ326940	Brackish Sediment	Conventional
MTZ1.2	99.92	*Streptomyces griseoaurantiacus* NBRC 15440	OQ326936	Brackish Sediment	Conventional
MTZ1.3	100.0	*Streptomyces griseoflavus* LMG 19344	OQ327036	Brackish Sediment	Conventional
MTZ3.25b	100.0	*Streptomyces griseoflavus* LMG 19344	OQ327033	Brackish water	Conventional
MTZ1.8	100.0	*Streptomyces griseoincarnatus* LMG 19316	OQ326965	Brackish Sediment	Conventional
MTZ3.19	100.0	*Streptomyces griseoincarnatus* LMG 19316	OQ326968	Brackish water	Conventional
MTZ3.5	100.0	*Streptomyces griseoincarnatus* LMG 19316	OQ326970	Brackish water	Conventional
MTZ1.13a	99.77	*Streptomyces halophytocola* KLBMP 1284	OQ326985	Brackish Sediment	Conventional
MTZ1.18	99.77	*Streptomyces halophytocola* KLBMP 1284	OQ326967	Brackish Sediment	Conventional
MTZ1.5	99.92	*Streptomyces heliomycini* NBRC 15899	OQ326934	Brackish Sediment	Conventional
ICT_C11.1a	99.46	*Streptomyces intermedius* NBRC 13049	OQ326982	Brackish Sediment	iChip
ICT_C11.1b	99.39	*Streptomyces intermedius* NBRC 13049	OQ326992	Brackish Sediment	iChip
MTZ1.9	99.43	*Streptomyces intermedius* NBRC 13049	OQ326952	Brackish Sediment	Conventional
ICT_B6.4	99.53	*Streptomyces karpasiensis* K413	OQ326989	Brackish Sediment	iChip
MTZ2.3	100.0	*Streptomyces lienomycini* LMG 20091	OQ326935	*Ulva* sp.	Conventional
ICT_C8.2	99.84	*Streptomyces malaysiense* MUSC 136	OQ327004	Brackish Sediment	iChip
MTZ2.4	99.31	*Streptomyces marokkonensis* Ap1	OQ326974	*Ulva* sp.	Conventional
MTZ3.2	99.31	*Streptomyces marokkonensis* Ap1	OQ326973	Brackish water	Conventional
MTZ3.1	100.0	*Streptomyces meridianus* MTZ3.1	MZ475064	Brackish water	Conventional
MTZ3.4	99.29	*Streptomyces mexicanus* CH-M-1035	OQ326942	Brackish water	Conventional
MTZ1.10	99.92	*Streptomyces qinglanensis* 172205	OQ326955	Brackish Sediment	Conventional
ICT_B12.1	100.0	*Streptomyces setonii* NRRL ISP-5322	OQ326979	Brackish Sediment	iChip
ICT_B12.2	100.0	*Streptomyces setonii* NRRL ISP-5322	OQ327016	Brackish Sediment	iChip
MTZ2.6	99.84	*Streptomyces sparsogenes* ATCC 25498	OQ326977	*Ulva* sp.	Conventional
MTZ3.20	99.68	*Streptomyces speibonae* NRRL B-24240	OQ326945	Brackish water	Conventional
MTZ3.7	99.72	*Streptomyces violaceorubidus* LMG 20319	OQ326941	Brackish water	Conventional

MTZ3.17	100.0	*Streptomyces xinghaiensis* S187	OQ326963	Brackish water	Conventional
MTZ3.3	99.84	*Streptomyces yanii* NBRC 14669	OQ327034	Brackish water	Conventional

Table S2 – Matches of putatively detected non-identified molecules in HPLC/HRMS in the DNP

Molecular Formula	Hits in the DNP	Hit 1	Hit 2	Hit 3	Hit 4	Hit 5	Hit 6	Hit 7	Hit 8	Hit 9	Hit 10	Hit 11	Hit 12
$C_8H_8N_2S$	0	—	—	—	—	—	—	—	—	—	—	—	—
$C_{10}H_{16}NO_5$	0	—	—	—	—	—	—	—	—	—	—	—	—
$C_{10}H_{19}NO_5$	0	—	—	—	—	—	—	—	—	—	—	—	—
$C_{12}H_{16}N_2O_2$	0	—	—	—	—	—	—	—	—	—	—	—	—
$C_{13}H_{15}N_2O_3$	0	—	—	—	—	—	—	—	—	—	—	—	—
$C_{14}H_{31}NO$	0	—	—	—	—	—	—	—	—	—	—	—	—
$C_{16}H_{34}O_6S$	0	—	—	—	—	—	—	—	—	—	—	—	—
$C_{18}H_{30}O_2$	0	—	—	—	—	—	—	—	—	—	—	—	—
$C_{19}H_{32}O_3S$	0	—	—	—	—	—	—	—	—	—	—	—	—
$C_{21}H_{30}O_5$	0	—	—	—	—	—	—	—	—	—	—	—	—
$C_{21}H_{33}N_3O_3$	0	—	—	—	—	—	—	—	—	—	—	—	—
$C_{24}H_{41}N_3O_7$	0	—	—	—	—	—	—	—	—	—	—	—	—
$C_{25}H_{44}O_3S_2$	0	—	—	—	—	—	—	—	—	—	—	—	—
$C_{25}H_{49}N_5O$	0	—	—	—	—	—	—	—	—	—	—	—	—
$C_{25}H_{50}N_{10}$	0	—	—	—	—	—	—	—	—	—	—	—	—
$C_{26}H_{42}O_5$	0	—	—	—	—	—	—	—	—	—	—	—	—
$C_{34}H_{42}N_4O_7$	0	—	—	—	—	—	—	—	—	—	—	—	—
$C_{49}H_{81}N_9O_9$	0	—	—	—	—	—	—	—	—	—	—	—	—
$C_{10}H_{10}O_4$	1	4-(2-Hydroxyphenyl)-4-oxobutanoic acid	—	—	—	—	—	—	—	—	—	—	—
$C_{12}H_{10}N_4O_2$	1	Lumichrome	—	—	—	—	—	—	—	—	—	—	—
$C_{12}H_{18}O_5$	1	Antibiotic SEN 366F	—	—	—	—	—	—	—	—	—	—	—
$C_{13}H_{23}NO_2$	1	Cornifronin B	—	—	—	—	—	—	—	—	—	—	—
$C_{17}H_{34}O_4$	1	Secotanikolide	—	—	—	—	—	—	—	—	—	—	—
$C_{18}H_{27}NO_5$	1	9-(4-Aminophenyl)-3,7-dihydroxy-2,4,6-trimethyl-9-oxononanoic acid	—	—	—	—	—	—	—	—	—	—	—
$C_{18}H_{32}O_4$	1	Punaauic acid	—	—	—	—	—	—	—	—	—	—	—
$C_{22}H_{30}O_5$	1	Bendigole D	—	—	—	—	—	—	—	—	—	—	—
$C_8H_5NO_3$	1	6-Hydroxyisatin	—	—	—	—	—	—	—	—	—	—	—
$C_{12}H_{11}NO_2S$	2	Brassicanal B	Chuangxinmycin	—	—	—	—	—	—	—	—	—	—
$C_{13}H_{14}N_2O_3$	2	Antibiotic DC 81	Caerulomycin G	—	—	—	—	—	—	—	—	—	—
$C_{13}H_{20}O_2$	2	2-Butyl-5-propylresorcinol	2-Hexyl-5-methylresorcinol	—	—	—	—	—	—	—	—	—	—
$C_{15}H_{20}N_2O_4$	2	Bripiodionene	Epiderstatin	—	—	—	—	—	—	—	—	—	—
$C_{18}H_{16}N_2$	2	Vibrindole A	1,2-Di(1H-indol-3-yl)ethane	—	—	—	—	—	—	—	—	—	—
$C_{20}H_{34}N_2O_6$	2	Eponemycin	Heronapyrrole A	—	—	—	—	—	—	—	—	—	—
$C_{22}H_{16}O_6$	2	Resistomycin	Bisanhydroaklavinone	—	—	—	—	—	—	—	—	—	—
$C_{26}H_{42}N_4O_6$	2	Rakicidin C	Cepafungin III	—	—	—	—	—	—	—	—	—	—
$C_{28}H_{37}N_3O_6$	2	Novel virginiamycin	Tyropeptin A	—	—	—	—	—	—	—	—	—	—
$C_8H_{13}NO_3$	2	Desmodilactone	*N*-Butanoylhomoserine lactone	—	—	—	—	—	—	—	—	—	—
$C_9H_{12}N_2O_2S$	2	3-(Acetylamino)-*N*-(2-thienyl)propanamide	S-(2-Acetamidoethyl) 1H-pyrrole-2-carbothioate	—	—	—	—	—	—	—	—	—	—
$C_{10}H_{11}NO_4$	3	2-Amino-3-(3-formyl-4-hydroxyphenyl)propanoic acid	2-Acetamido-3,4-cresotic acid	4-Hydroxy-4-(4-nitrophenyl)-2-butanone	—	—	—	—	—	—	—	—	—
$C_{10}H_{16}N_2O_2$	3	Amiclenomycin	2-Amino-*N*-[(2-cyclohexen-1-yl)acetyl]acetamide	Cyclo(prolylvalyl)	—	—	—	—	—	—	—	—	—
$C_{10}H_9NO$	3	4-Hydroxy-2-methylquinoline	2-Quinolinemethanol	4-Quinolinemethanol	—	—	—	—	—	—	—	—	—
$C_{11}H_{11}NO_3$	3	3-Hydroxy-3-(2-oxopropyl)-2-indolinone	3-(2,3-Dihydroxypropanoyl)-1H-indole	3-(3-Indolyl)lactic acid	—	—	—	—	—	—	—	—	—
$C_{11}H_{14}O_4$	3	2,3,6,7,8,9b-Hexahydro-1-hydroxy-1H-cyclopenta[d]pyrano[2,3-b]pyran-9(4aH)-one	1-O-(Phenylacetyl)glycerol	Xialenon B	—	—	—	—	—	—	—	—	—
$C_{11}H_{15}NO_2$	3	*N*-Propanoyltyramine	*N*-(2-Hydroxy-3-phenylpropyl)acetamide.	Ethyl *N*-(2-phenylethyl)carbamate	—	—	—	—	—	—	—	—	—
$C_{14}H_{17}NO_2$	3	2-Pentyl-3,4-quinolinediol	2-Pentyl-4(1H)-quinolinone	Xenocyloin A	—	—	—	—	—	—	—	—	—
$C_{10}H_{13}NO$	4	Dihydroabikoviromycin	Streptazone D	Kitasatodine	*N*-(2-Phenylethyl)acetamide	—	—	—	—	—	—	—	—
$C_{12}H_{14}O_4$	4	Palatinol A	Monobutyl phthalate	4,8-Dihydroxy-3-hydroxymethyl-4-methyl-1-tetralone	2-Hydroxy-6-(3-oxobutyl)phenyl acetate. 4-(2-Acetoxy-3-hydroxyphenyl)-2-butanone	—	—	—	—	—	—	—	—
$C_{12}H_{20}O_3$	4	Alkyldihydropyrone D	Alkyldihydropyrone C	MKN 003B	10-Hydroxy-2-dodecen-4-olide	—	—	—	—	—	—	—	—
$C_{15}H_{16}O_5$	4	Pentalenolactone	Pentalenolactone A	Pentalenolactone A	Pentalenolactone P	—	—	—	—	—	—	—	—
$C_{15}H_{18}O_6$	4	Pentalenolactone H	Pentalenolactone O	Aspvanicin B	Aspvanicin A	—	—	—	—	—	—	—	—
$C_{18}H_{30}O_3$	4	9-Hydroxy-10,12,15-octadecatrienoic acid	Pseudopyronine B	Photopyrone D	13-Hydroxy-11-octadecen-9-ynoic acid	—	—	—	—	—	—	—	—
$C_7H_{11}NO_3$	4	Antibiotic Tu 1718A	Furanomycin	5,6,7,8-Tetrahydro-5,7-dihydroxy-1H-azocin-2-one	*N*-Acetyl-4-hydroxyvaline lactone	—	—	—	—	—	—	—	—
$C_{10}H_9NO_2$	5	2,4-Dihydroxy-3-methylquinoline	IAA	4-Methoxy-1(2H)-isoquinolinone	3-Methyl-1H-indole-2-carboxylic acid	Sporazepine	—	—	—	—	—	—	—
$C_{12}H_{22}O_3$	5	2,5-Dideoxy-2-(5-methylhexyl)-arabinonic acid	3-Hydroxy-5-dodecenoic acid	Nesterenkoniane	Streptoketol B	11,12,13-Trinor-1,5,8-eudesmanetriol	—	—	—	—	—	—	—
$C_{20}H_{22}O_6$	5	Elmycin E	Pinoresinol	Epipinoresinol	Kiamycin B	6a,7,12,12a-Tetrahydro-6a-hydroxy-8-O-methyltetrangomycin	—	—	—	—	—	—	—
$C_{10}H_{11}NO_3$	6	*N*-Propanoylanthranilic acid	*N*-[2-(4-Hydroxyphenyl)-2-oxoethyl]acetamide	Yanglingmycin	Nocazoline A	Isoferulamide	Madurastatin B3	—	—	—	—	—	—
$C_{13}H_{22}O_4$	6	Dihydro-4-(hydroxymethyl)-3-(6-methyl-1-oxoheptyl)-2(3H)-furanone	Ieodomycin A	7-Ethyl-4,9-dihydroxy-7-undecene-3,6-dione	7-Ethyl-4,10-dihydroxy-7-undecene-3,6-dione	5-(6,7-Dihydroxy-6-methyloctyl)-2(5H)-furanone	5-(4,6-Dihydroxyoctyl)-2(5H)-furanone	—	—	—	—	—	—
$C_{12}H_{12}N_2O_2$	7	*N*-[2-(1H-Indol-3-yl)-2-oxoethyl]acetamide	5-(2-Aminobenzoyl)-2-ethyloxazole	Sannanine	2,3-Dimethyl-8-(methylamino)-5,6-quinolinedione	Caerulomycin F	Cyclo(alanyldehydrophenylalanyl)	Mansouramycin A	—	—	—	—	—
$C_{13}H_{22}O_3$	8	7-Ethyl-10-hydroxy-7-undecene-3,6-dione	7-Ethyl-4-hydroxy-7-undecene-3,6-dione	7-Ethyl-9-hydroxy-7-undecene-3,6-dione	10-Hydroxy-11-methyl-2-dodecen-4-olide	Acaterin	5-(7-Hydroxy-6-methyloctyl)-2(5H)-furanone	MKN 003C	Trihomononactic acid lactone	—	—	—	—
$C_{12}H_{18}O_3$	10	Dihydrocoronafacic acid	Ieodomycin B	Elijopyrone A	Violapyrone J	Germicidin F	Violapyrone O	Violapyrone A	Nocapyrone M	Nocardiatone C	Nocardiopyrone B	—	—

$C_{15}H_{24}O_3$	10	10,15-Dihydroxy-4-cadinen-3-one	Kandenol A	Violapyrone T	Nocapyrone H	Nocapyrone B	Nocapyrone R	Nocardiopyrone A	Photopyrone A	Presulficidin C	Streptoone C	—	—
$C_{11}H_{16}O_3$	12	Germicidin A	Isogermicidin A	4-Hydroxymucidone	10-Hydroxymucidone	Fugomycin	4-Hydroxy-4-methyl-2-(1-hexenyl)-2-butenolide	Loliolide	Isololiolide	7-Hydroxymucidone	Nocapyrone L	3-(1-Hydroxyhexyl)-5-methylene-2(5H)-furanone	Ulufuranol

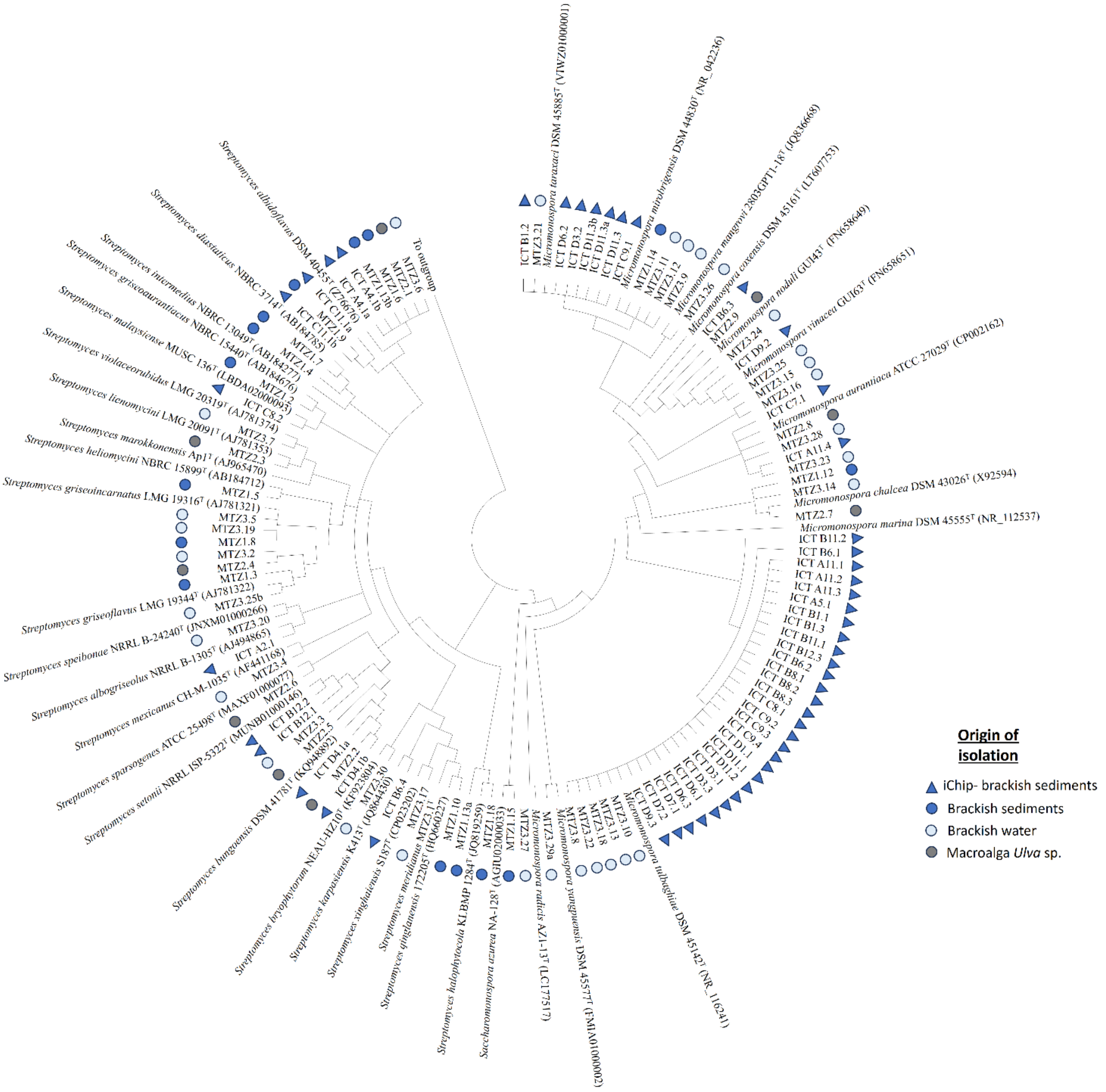

Supplementary Figure 1 - The 16S rRNA gene phylogeny tree with all isolated strains. The origin of isolation is of each isolate is defined by different markers and colours. The tree was inferred by using the Maximum Likelihood method and General Time Reversible model with 1000 bootstrap replications. The outgroup consists of the 16S rRNA gene sequences from *Stieleria sedimenti* ICT_E10.1^{T}, GenBank accession number OL684514.

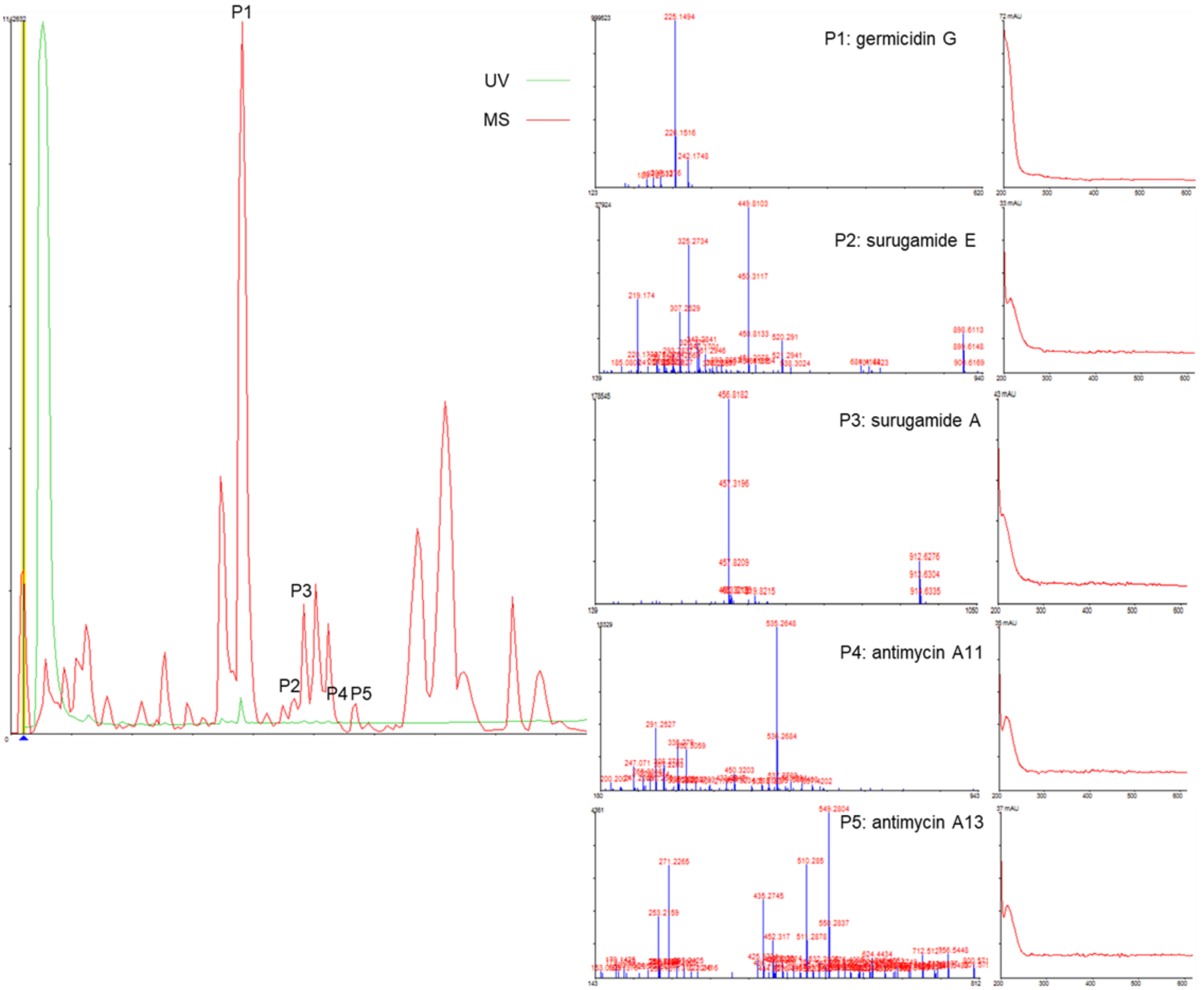

Supplementary Figure 2 - LC-MS chromatogram of strain ICT_A2.1 extract, (B) mass and (C) UV spectra of germicidin G, surugamides A and E and antimycins A11 and A13 that were putatively detected in the extract.

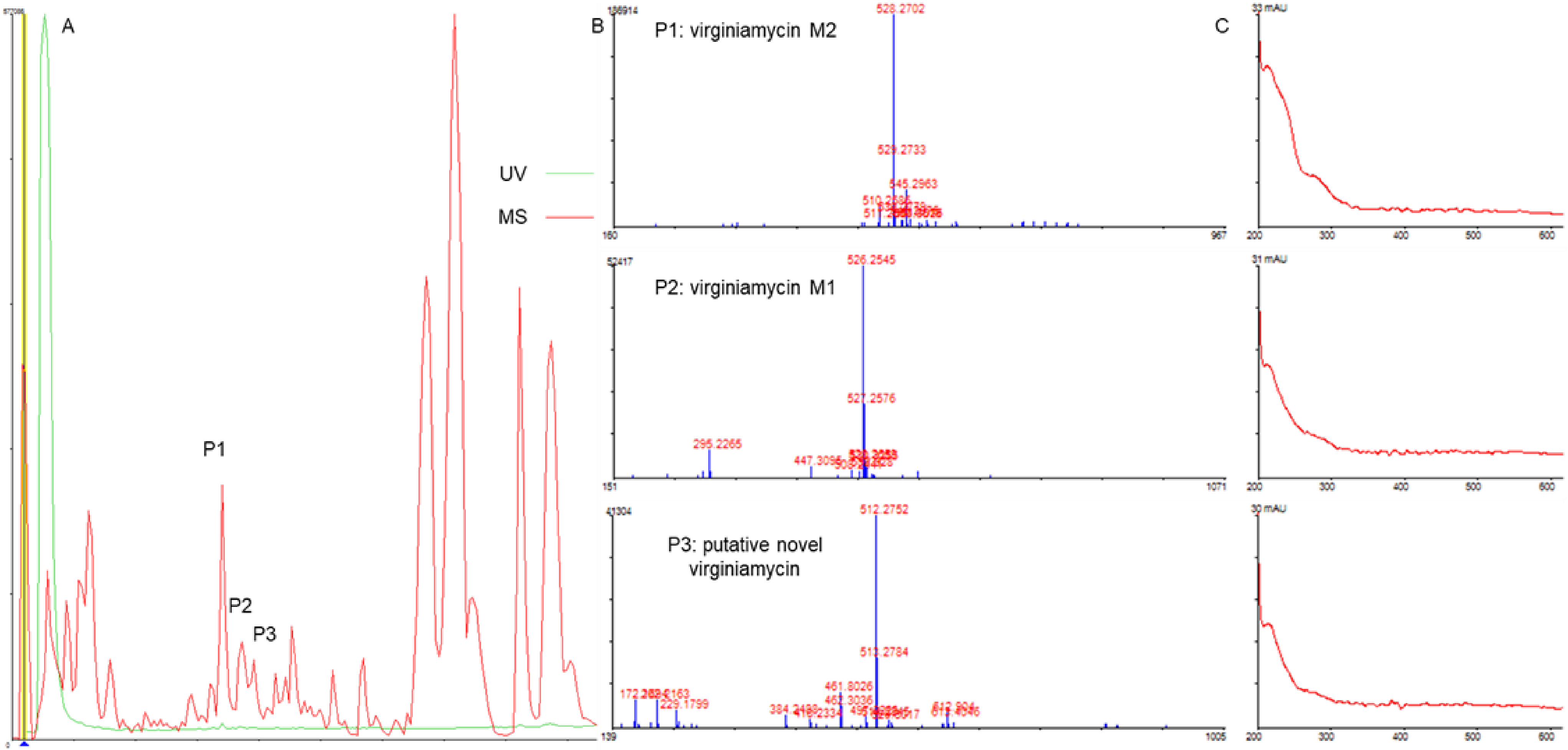

Supplementary Figure 3 – (A) LC-MS chromatogram of strain MTZ2.1 extract, (B) mass and (C) UV spectra of virginiamycin M1, virginiamycin M2 and the novel virginiamycin that were putatively detected in the extract.
